# BTP2, a store-operated calcium channel inhibitor, attenuates morphine antinociceptive tolerance in rats

**DOI:** 10.3389/fnins.2026.1758352

**Published:** 2026-04-01

**Authors:** Weibo Xiao, Feng Xiao, Yingying Zhang, Lei Zeng

**Affiliations:** 1 Department of Anesthesiology, Loudi Central Hospital, Loudi, China; 2 Department of Pediatric Surgery, Loudi Maternal and Child Health Care Hospital, Loudi, China; 3 Department of Neurology, Loudi Central Hospital, Loudi, China

**Keywords:** BTP2, ERK, GFAP, morphine, store-operated calcium channels, tolerance

## Abstract

**Introduction:**

Morphine antinociceptive tolerance remains a critical problem in the clinical management of pain. Spinal cord glial cell activation and neuroinflammation appear to play a crucial role in the development and maintenance of this tolerance. BTP2, a potent store-operated calcium channel inhibitor, has anti-inflammatory properties in the central nervous system. This study aimed to investigate the effect of BTP2 on the development of morphine antinociceptive tolerance and glial cell-derived pro-inflammatory cytokines production by chronic morphine treatment.

**Methods:**

A rat model of morphine antinociceptive tolerance was made by intrathecal injection of morphine (15 μg/d). Two separate studies were conducted: Firstly, to investigate whether BTP2 could attenuate the development of tolerance, BTP2 (2 and 10 nmol) was given intrathecally 30 min before each intrathecal delivery of morphine for consecutive 7 days. Secondly, to investigate whether BTP2 could reverse the established tolerance, BTP2 administration was initiated on day 8 after 7 days of morphine treatment and continued for 4 days.

**Results:**

The results showed that BTP2 not only attenuated the development of morphine tolerance but also partially reversed the established tolerance. Immunohistochemistry revealed that chronic morphine-induced activation of astrocytes in the spinal cord, while BTP2 was shown to suppress the activation of astrocytes. Moreover, the administration of BTP2 alleviated the activation of astrocytic ERK and the production of proinflammatory cytokines (e.g., TNF-α and Il-1β) in the spinal cord.

**Discussion:**

These findings suggest that BTP2 can be a potential therapeutic drug for morphine antinociceptive tolerance, and the store-operated calcium channel may play an important role in morphine antinociceptive tolerance.

## Introduction

Morphine and other opioids are the most effective analgesic drugs that are used to relieve patients with moderate to severe pain, including cancer pain and other chronic pain conditions. However, their analgesic effects are greatly reduced by the development of tolerance after repeated administration, thereby requiring larger doses for the maintenance of analgesia, which elevate the risk of developing side effects, such as constipation, urinary retention, and respiratory depression, thus hindering their clinical applications (Benyamin et al., 2008; Morrone et al., 2017).

Mounting evidence indicates that glia cell-derived neuroinflammation in the central nervous system (CNS) plays a crucial role in the development and maintenance of morphine analgesic tolerance (Eidson and Murphy, 2019; Watkins et al., 2009). Glia cells, both microglia and astrocytes, are activated following chronic morphine administration and release a series of pro-inflammatory cytokines, including interleukin-1β (IL-1β), IL-6, and tumor necrosis factor α (TNF-α) (Raghavendra et al., 2002; Wang et al., 2009), and the pro-inflammatory cytokines could enhance the excitability of spinal cord dorsal horn neurons (Hutchinson et al., 2011). Moreover, inhibition of glial activation or antagonizing the activity of glial proinflammatory cytokines can markedly relieve morphine tolerance (Raghavendra et al., 2004; Hutchinson et al., 2008; Shen et al., 2012). These findings suggest that suppression of glial activation by inhibiting the production of proinflammatory cytokines with great promise for alleviating morphine tolerance.

Astrocytes are the most abundant glial cells in the CNS. It is estimated that every single astrocyte can enwrap on average 4 to 8 neuronal somata and contact up to 300–600 neuronal dendrites in rodents (Halassa et al., 2007), providing an ability to maintain and regulate the chemical environment of neurons. The activity of astrocytes largely depends on the intracellular Ca^2+^ level. It has been shown that astrocytes could respond to a wide range of extracellular stimuli by raising cytosolic Ca^2+^ (Khakh and McCarthy, 2015; Agulhon et al., 2008). One of the key regulatory mechanisms of Ca^2+^ homeostasis in astrocytes is the store-operated calcium entry (SOCE) (Kwon et al., 2017; Papanikolaou et al., 2017; Okubo et al., 2020), which is mediated by store-operated calcium channels (SOCCs), and SOCE has also been reported to occur in astrocytes (Golovina, 2005). There are two essential components of SOCCs: the plasmalemma channels Orai proteins and the endoplasmic reticulum Ca^2+^ sensors stromal interaction molecule (STIM) proteins. SOCCs are highly Ca^2+^-selective cation channels that can be activated by the depilation of endoplasmic reticulum Ca^2+^ stores (Prakriya and Lewis, 2015). One of the important functions of SOCE in astrocytes is regulating proinflammatory cytokine production. It has been shown that BTP2 (also known as YM-58483), a potent SOCCs blocker, could block SOCE and reduce TNF-α and IL-6 production from spinal astrocytes (Gao et al., 2016). Consistent with its anti-inflammatory effect, previous work has demonstrated that intrathecal administration of BTP2 could reduce spinal proinflammatory cytokine (e.g., IL-1β, TNF-α, and PGE2) expression in a rat neuropathic pain model with fifth lumbar (L5) spinal nerve ligation (Qi et al., 2016). However, the effects of BTP2 on morphine analgesic tolerance have not been examined.

Given that morphine analgesic tolerance is associated with the neuroinflammatory response induced by activated glial cells in the CNS, and SOCCs have a crucial role in this response, we hypothesized that SOCCs inhibitors have an effective function on morphine analgesic tolerance. In this study, two separate experiments were conducted to explore the effects of BTP2, a pharmacological inhibitor of SOCCs, on the development and reversal of morphine analgesic tolerance in rats, thereby providing *in vivo* pharmacological evidence for the potential involvement of SOCC signaling in these processes.

## Materials and methods

### Animals

Adult male Sprague–Dawley rats weighing 180–220 g were obtained from Hunan SJA Laboratory Animal Co., Ltd. (Changsha, China). Rats were individually housed in plastic cages on a 12 h light/dark cycle with a standard rat diet. All experimental procedures were performed according to the guidelines of the International Association for the Pain Study and approved by the ethics committee of the Second Xiangya Hospital.

### Intrathecal catheter implantation and drug administration

For intrathecal (i.t.) drug delivery, rats were intrathecally implanted with polyurethane (PE) catheters as we described previously (Qi et al., 2016). Briefly, under pentobarbital anesthesia, a PE-10 tube was implanted into the lumbar subarachnoid space and extended to the lumbar enlargement through the gap between the vertebrae L5 and L6. The exterior end of the tube was exited at the back of the neck by tunneling subcutaneously. All the animals were allowed to recover for 5–7 days and habituated to the test environment daily for at least 3 days before experiments.

Drugs were injected through the i.t. catheter in a total volume of 10 μL, followed by a flush of 15 μL of saline to complete delivery into the subarachnoid space. Morphine sulfate (Shenyang First Pharmaceutical Factory, China) was dissolved in saline at a concentration of 1.5 μg/μL. According to the previously described method (Qi et al., 2016), BTP2 (Tocris Bioscience, MN, USA) was dissolved in DMSO as a stock solution and then further diluted to 2% DMSO/cremophor EL/saline solution at concentrations of 200 μM and 1,000 μM. The doses of BTP2 (2 and 10 nmol) were selected based on previous studies demonstrating their efficacy in attenuating neuropathic pain and spinal inflammatory responses without affecting baseline nociception (Qi et al., 2016; Gao et al., 2015).

### Induction of morphine tolerance and behavioral test

Tolerance to morphine analgesic effect was induced by a daily intrathecal injection of morphine (15 μg/day) for 7 consecutive days as described previously (Wang et al., 2010). Analgesic effects of morphine were tested by paw withdrawal latency both before and 30 min after morphine administration. For the paw withdrawal latency test, a radiant heat beam was focused on the plantar surface of the hind paw using a plantar test device (37370, Ugo Basile, Italy). The baseline latency was set to 10–12 s with a maximum of 20 s as a cut-off time to avoid potential tissue damage. The latencies were averaged over three trials with an intertrial interval of 5 min. The percentage of maximal possible antinociceptive effect (%MPE) was calculated according to the formula: %MPE = [(post-drug latency − baseline latency)/(cut-off time − baseline latency)] × 100.

### Experimental design

To explore whether BTP2 would attenuate the development of tolerance to morphine antinociception in rats, BTP2 (2 and 10 nmol) was intrathecally administered 30 min before each morphine treatment from day 1 to day 7. The behavioral test was recorded from day 1 to 7, and the lumbar spinal cord (L4-L6) tissues were harvested on day 7 after the behavioral test.

To investigate the effect of BTP2 on morphine antinociceptive tolerance in rats with established tolerance, morphine was intrathecally administered from day 1 to 11 for 11 consecutive days. BTP2 (2 and 10 nmol) was given 30 min before each morphine treatment from day 8 to day 11 for 4 consecutive days. An additional control group received BTP2 (10 nmol) alone from day 8 to 11, which showed no significant effect on baseline paw withdrawal latency. The behavioral test was recorded from day 1 to 11, and the lumbar spinal cord (L4-L6) tissues were harvested on day 11 after the behavioral test.

### Immunofluorescence

Animals were perfused transcardially with 0.9% saline, followed by a fixative containing 4% paraformaldehyde in 0.1 M phosphate buffer (PH 7.4). After perfusion, lumbar spinal cords (L4-L5) were quickly removed and postfixed in 4% paraformaldehyde at 4 °C for 4 h and then sequentially transferred to 30% sucrose at 4 °C until immersion and embedded in O.C.T. compound. The spinal cords were cut into 20-μm-thick slices at −20 °C using a cryostat (CM1950, Leica, Germany). Sections were blocked with normal donkey serum at a concentration of 10% in 0.3% Triton X-100 in PBS for 1 h at room temperature and incubated overnight at 4 °C with the following primary antibodies: mouse anti-GFAP (1:2000, Millipore), rabbit anti-p-ERK (1:1000, CST), mouse anti-NeuN (1:2000, Millipore), goat anti-IBA1 (1:500, Abcam). After 3 washes in PBS, the sections were incubated with Alexa 488- or Alexa 594-conjugated secondary antibodies (1:1000, Invitrogen, USA) in the blocking solution for 1 h at room temperature in the dark. After final washing in PBS, sections were mounted with VECTASHIELD mounting medium with DAPI (Vector Laboratories, USA). Images were captured using a fluorescence microscope (Olympus BX51, Japan).

### Western blot analysis

Animals were decapitated after behavioral tests. The lumbar spinal cord was removed. Tissue samples were homogenized in RIPA buffer (Beyotime, China) on ice supplemented with protease and phosphatase inhibitor cocktails (Roche, USA). The lysed tissues were centrifuged at 12,000 × g (4 °C) for 10 min, and the protein concentrations were measured by a BCA protein assay kit. Protein solutions were mixed with loading buffer and denatured at 95 °C for 5 min, and the protein samples were electrophoresed in a 10% SDS-PAGE and transferred to nitrocellulose (NC) membranes (Millipore, USA) using a Bio-Rad Mini Trans-Blot apparatus. The membranes were blocked with 5% non-fat milk in Tris-buffered saline with Tween 20 (TBST) for 1 h at room temperature and incubated at 4 °C overnight with the following primary antibodies: mouse anti-GFAP (1:2000, Millipore), rabbit anti-p-ERK (1:1000, CST), rabbit anti-ERK (1:10000, CST), and mouse anti-*β*-Actin (1:5000, CMCTAG, WI, USA). The membranes were washed three times in TBST buffer and incubated for 1 h at room temperature with horseradish peroxidase-conjugated secondary antibodies, then detected using enhanced ECL solution (Millipore, USA). The density of protein bands was quantified by Image-Pro Plus 6.0 analysis software (Media Cybernetics, USA).

### ELISA

Animals were deeply anesthetized with isoflurane after behavioral testing, and the spinal cords (L4-L6) were removed. Tissue samples were weighed, homogenized, and centrifuged. The supernatants were collected, and the concentrations of IL-1β and TNF-α were measured using ELISA kits (R&D Systems, Minneapolis, MN, USA) according to the manufacturer’s instructions.

### Statistical analysis

Results were presented as means ± SEM. Behavioral data were analyzed through a two-way repeated ANOVA followed by Tukey’s *post hoc* test. Data from western blot and immunofluorescence were compared using one-way ANOVA followed by Tukey’s or Dunn’s *post hoc* test. Statistical analyses were performed with GraphPad Prism 6 software (La Jolla, CA), and the error probabilities of *p* < 0.05 were considered statistically significant.

## Results

### Preemptive intrathecal BTP2 delayed the development of morphine analgesic tolerance

**Figure 1:**
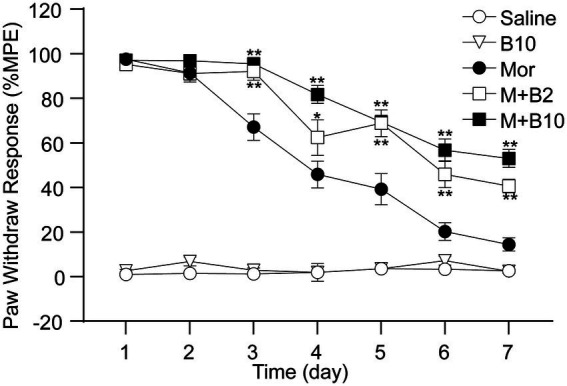
Inhibition of the development of morphine analgesic tolerance by co-administration of a SOCCs inhibitor BTP2. Paw withdrawal latency to thermal stimuli was tested daily before and 30 min after injection from day 1 following a daily intrathecal delivery of saline 10 μL (Saline), BTP2 10 nmol (B10), morphine 15 μg (Mor), or morphine with BTP2 2 nmol (M + B2) or 10 nmol (M + B10) for 7 days. BTP2 was given 30 min before morphine treatment. %MPE: percentage of maximal possible antinociceptive effect. Data were expressed as the mean ± SEM (*n* = 6–7 per group). *p < 0.05, **p < 0.01 compared with morphine group at identical time point.

Intrathecal morphine (15 μg/day) produced a maximal antinociceptive effect on day 1. Repeated 7-day daily intrathecal delivery of morphine led to decreased paw-withdrawal response time-dependently, which reflects the development of tolerance to morphine analgesia (Figure 1). To determine whether SOC channels play a role in morphine analgesic tolerance, we examined the effect of BTP2, a potent SOC channel blocker, on rats with morphine tolerance. BTP2 was intrathecally administered 30 min before each morphine injection. As shown in Figure 1, a 7-day treatment with saline or BTP2 (10 nmol/day) alone did not change baseline latency; however, the co-treatment with BTP2 (2 and 10 nmol/day) attenuated the development of antinociceptive tolerance (*p* < 0.01, compared with the morphine group), suggesting BTP2 retained the antinociceptive effect of morphine.

### BTP2 partially reverses the established tolerance to morphine analgesia

**Figure 2:**
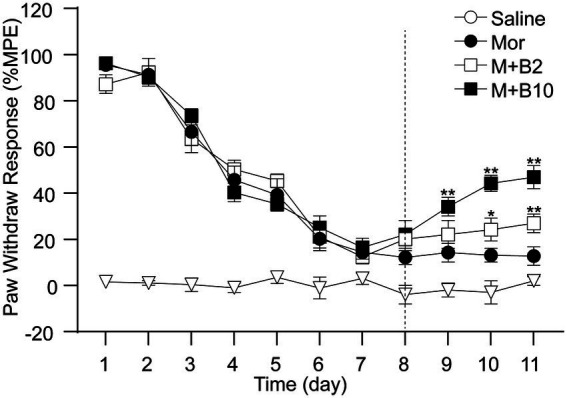
BTP2 reversed the established morphine analgesic tolerance. BTP2 administration was initiated on day 8 (indicated by the dotted line) after 7 days of morphine treatment and continued for 4 days. In morphine with BTP2 groups (M + B2 and M + B10), morphine still produced a significant analgesic effect from day 9 compared with the morphine group (Mor). Data are expressed as the mean ± SEM (*n* = 6–7 per group). *p < 0.05, **p < 0.01 compared with morphine group at identical time point.

Since the intrathecal pretreatment of BTP2 significantly delayed the development of morphine analgesic tolerance, to investigate whether BTP2 could reverse the established tolerance to morphine analgesia, BTP2 was administrated after 7-day morphine treatment. As shown in Figure 2, BTP2 was given intrathecally 30 min before morphine injection on day 8 and continued for 4 consecutive days. Our data showed that morphine produced significant analgesia in groups treated with BTP2 than in the group treated with morphine alone. These results demonstrate that BTP2 could reverse the established morphine tolerance.

### Effects of BTP2 on spinal astrocytes activation in morphine antinociceptive tolerance

**Figure 3:**
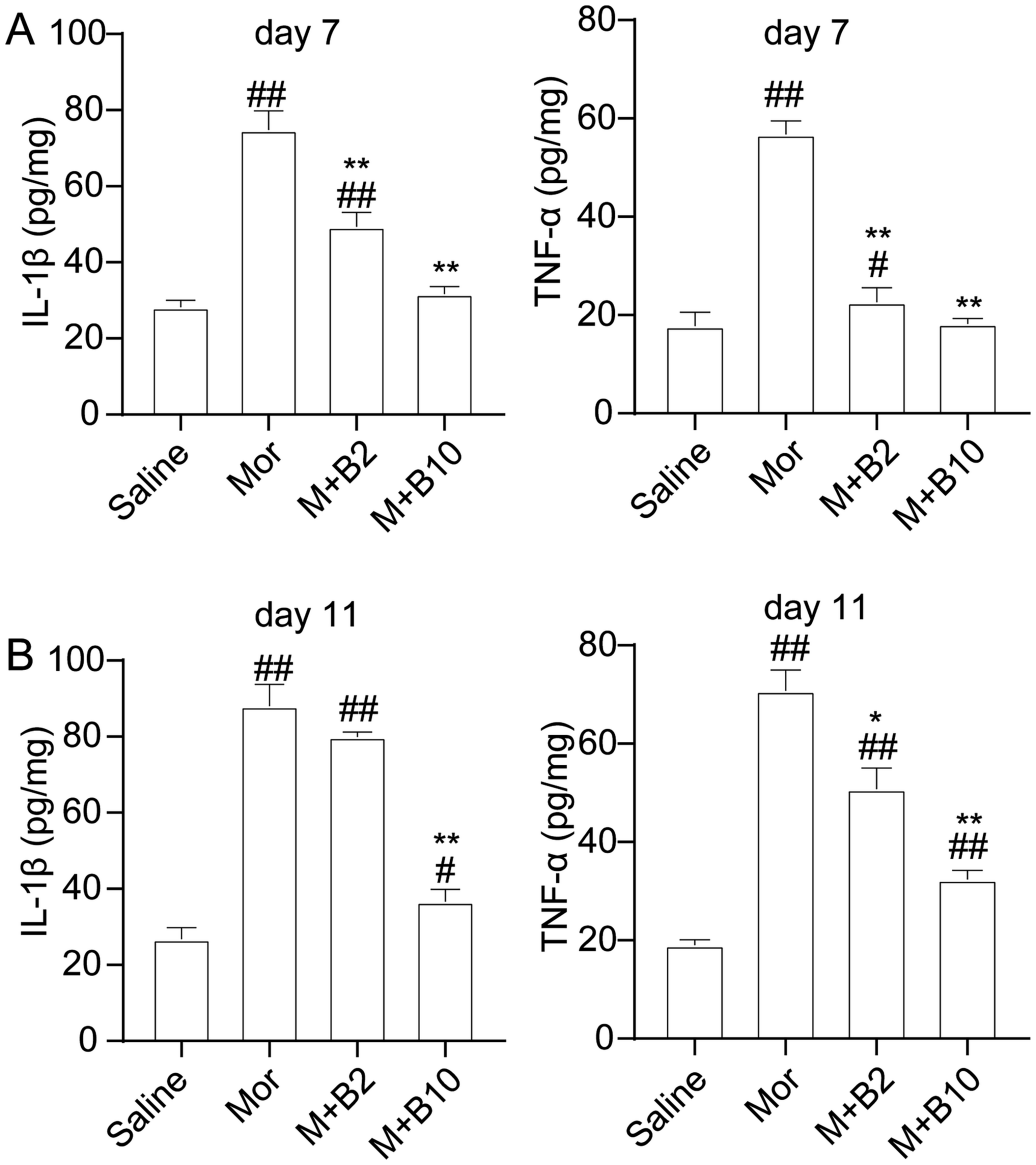
Effect of intrathecal BTP2 on chronic morphine-induced spinal astrocytes activation. **(A)** BTP2 attenuated spinal astrocytes activation in rats with tolerance development. Immunohistochemistry revealed an increased GFAP (astrocyte marker) expression in the spinal cord dorsal horn following intrathecal delivery of morphine (15 μg) for 7 days compared with saline groups. Co-administration of BTP2 10 nmol with morphine (15 μg) for 7 days significantly decreased the activation of spinal astrocytes. **(B)** BTP2 decreased spinal astrocytes activation in rats with established morphine tolerance. An increased GFAP expression in the spinal cord dorsal horn following intrathecal delivery of morphine (15 μg) for 11 days compared with saline groups. Co-administration of BTP2 10 nmol with morphine (15 μg) on day 8 and continued for 4 days significantly reduced the activation of spinal astrocytes. Data were expressed as the mean ± SEM (*n* = 6–7 per group). *p < 0.05 compared with morphine group, #*p* < 0.05 compared with saline group. Scale bar, 100 m.

To determine the effect of BTP2 on the activation of astrocytes in the lumbar spinal cord, immunohistochemistry was carried out to detect the expression level of the astrocyte-specific marker GFAP. As shown in Figure 3, after 7 days of morphine treatment, the spinal astrocytes were significantly activated as manifested by hypertrophic morphology and increased immunoreactivity of GFAP. However, a co-treatment with BTP2 prevented chronic morphine-induced astrocyte hypertrophy and GFAP intensity (A). BTP2 also inhibited the expression of GFAP in established morphine-tolerant rats on day 11 (B). These results suggest BTP2 could attenuate astrocytes activation in the spinal cord in morphine-tolerant rats.

### BTP2 reduces the activation of ERK in the spinal cord of morphine antinociceptive tolerance rats

**Figure 4:**
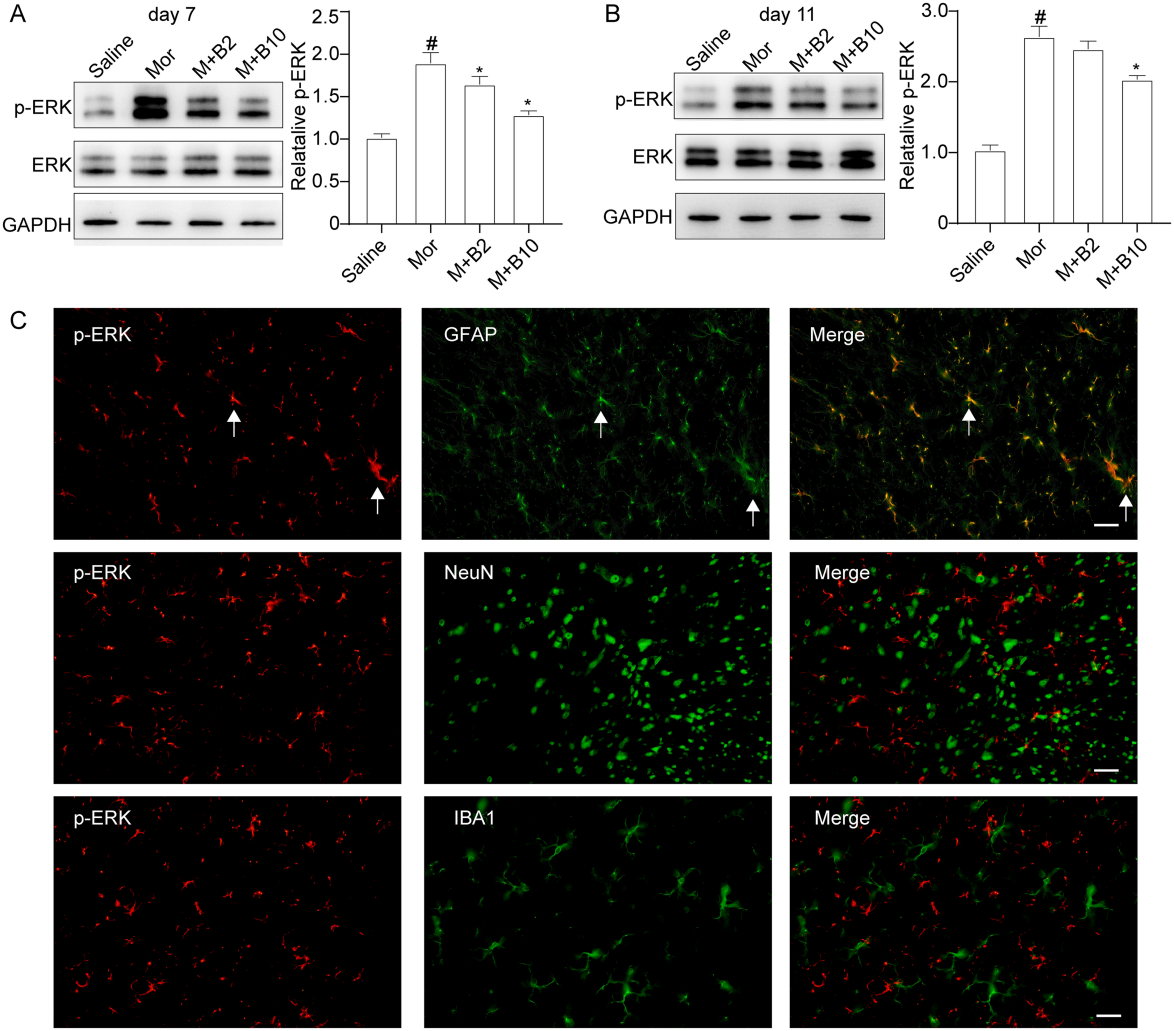
Effect of intrathecal BTP2 on chronic morphine-induced spinal ERK activation. **(A)** BTP2 attenuated spinal ERK activation in rats with tolerance development. The spinal protein expression of p-ERK was assessed by western blot. Intrathecal administration of morphine (15 μg) for 7 days resulted in an increase of p-ERK in the spinal cord compared with saline groups. Co-administration of BTP2 2 or 10 nmol with morphine (15 μg) for 7 days decreased the p-ERK. **(B)** BTP2 reduced spinal ERK activation in rats with established tolerance. An increased p-ERK expression following intrathecal delivery of morphine (15 μg) for 11 days compared with saline groups. BTP2 2 or 10 nmol administration beginning on day 8 and lasting for the next 4 days resulted in a reversal of the increase of p-ERK expression compared with the morphine group (Mor). **(C)** Cellular localization of p-ERK in the spinal dorsal horn following 7-day morphine treatment. Double immunofluorescence reveals that revealed p-ERK (red) is co-localized with GFAP (astrocyte marker, green, arrows) but not with IBA1 (microglia marker, green) or NeuN (neuronal marker, green). Data were expressed as the mean ± SEM (*n* = 6–7 per group). *p < 0.05 compared with morphine group, #*p* < 0.05 compared with saline group. Scale bar, 50 μm.

It has been shown that ERK was found to be activated in the spinal astrocyte by chronic morphine treatment, and BTP2 was reported to inhibit ERK activation. We tested whether BTP2 could inhibit ERK activation in the astrocytes of morphine tolerance rats. As shown in Figure 4, chronic treatment with morphine (15 μg/day for 7 days) induced a substantial increase in the expression of phosphor-ERK (p-ERK) in the spinal cord. However, BTP2 pretreatment (2 and 10 nmol/day) significantly reduced the activation of ERK (A). BTP2 also inhibited the activation of p-ERK in established morphine-tolerant rats on day 11 (B). Furthermore, double immunofluorescence revealed p-ERK was predominantly localization in astrocytes. These findings suggest that BTP2 can effectively inhibit chronic morphine-induced activation of ERK in astrocytes (C).

### BTP2 decreases the production of cytokines in the spinal cord in rats with morphine analgesic tolerance

**Figure 5:**
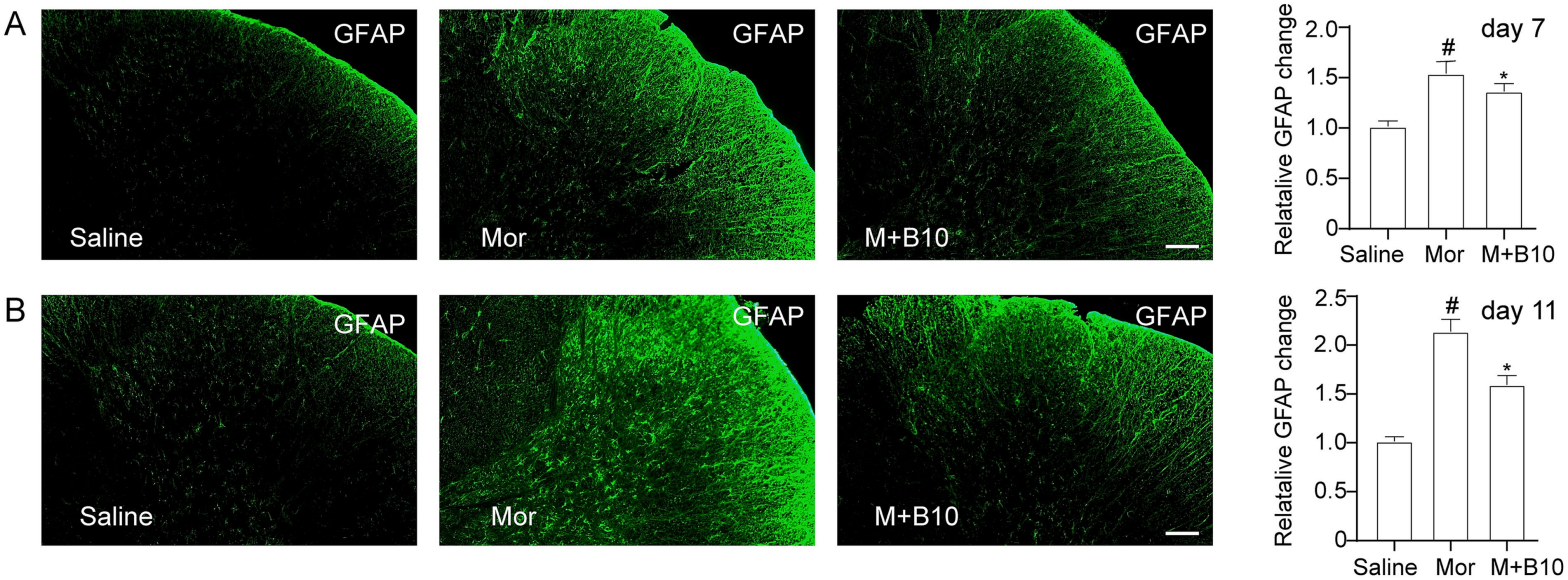
Effect of BTP2 on chronic morphine-induced spinal cytokine release. **(A)** ELISA data showed a significant increase of TNF-α and IL-1β levels in the spinal cord of rats intrathecally treated with morphine for 7 days. Co-administration of BTP2 2 or 10 nmol with morphine (15 μg) for 7 days prevented the increase in TNF-α and IL-1β levels. **(B)** BTP2 reduced spinal TNF-α and IL-1β production in rats with established tolerance on day 11. Data were expressed as the mean ± SEM (*n* = 6–7 per group). *p < 0.05, **p < 0.01 compared with morphine group, #*p* < 0.05, ##*p* < 0.01 compared with saline group.

Various pro-inflammatory cytokines, such as TNF-α and IL-1β, are expressed in the spinal cord and involved in the development and maintenance of tolerance to morphine analgesia. To investigate whether anti-tolerant actions of BTP2 are associated with proinflammatory cytokine levels, we measured the TNF-α and IL-1β levels in the lumbar spinal cord. Repeated administration of morphine for 7 days significantly increased the production of TNF-α and IL-1β in the spinal cord. However, both pretreatment and treatment with BTP2 dose-dependently decreased these pro-inflammatory cytokine levels (Figure 5A). BTP2 also reduced the levels of TNF-α and IL-1β in established morphine-tolerant rats on day 11 (Figure 5B). These findings strongly suggest that the positive effects of BTP2 in morphine-tolerant rats are associated with reductions in pro-inflammatory cytokine production.

## Discussion

The present study demonstrates that the SOCCs inhibitor BTP2 prevents the development of morphine antinociceptive tolerance and even partially reverses morphine antinociceptive tolerance once it has been previously established. Treatment with BTP2 attenuates spinal astrocyte marker GFAP immunoreactivity and suppresses repeated morphine-mediated astrocytic ERK activation. Moreover, BTP2 co-administration reduces the proinflammatory cytokines (TNF-α and IL-1β) release in the spinal cord of morphine-tolerant rats. This study provides the first *in vivo* evidence that inhibition of SOCCs produces a robust anti-neuroinflammatory response and enhances morphine’s analgesic effects in the tolerant rats, suggesting spinal astrocytic SOCCs may contribute to spinal neuroinflammatory consequences of long-term opioid analgesic treatment.

Morphine and other opioid analgesics are often the last resort for pain relief in many patients with severe or chronic pain, but their analgesic effects decrease after tolerance has been established. Numerous experimental evidences have shown that spinal cord glial cell activation, including microglia and astrocytes, plays a pivotal role in the development and maintenance of opioid tolerance (Sanna et al., 2019; Cao et al., 2016; Lin et al., 2015; Zhou et al., 2010). However, microglia and astrocytes may have different roles in morphine antinociceptive tolerance. It seems that microglia are mainly responsible for the initiation of morphine antinociceptive tolerance, while astrocytes are more important for the maintenance of it. Previous studies have reported that the microglia inhibitor, such as minocycline, could only prevent the development of morphine antinociceptive tolerance, but failed to reverse the established tolerance (Cui et al., 2008; Mika et al., 2009). On the contrary, inhibitors that could suppress microglia and astrocyte activation have been shown to with preventive and therapeutic activities (Li et al., 2015; Xu et al., 2015). It was previously found that BTP2 could suppress the activation of both microglia (Siddiqui et al., 2012; Ferreira and Schlichter, 2013) and astrocytes (Gao et al., 2016; Singaravelu et al., 2006). In the present study, we found that intrathecal treatment with BTP2 reverses not only the established morphine analgesic tolerance but also the activities of spinal astrocytes induced by chronic morphine administration, suggesting that inhibition of astrocytes may contribute to the effect of BTP2 on established morphine analgesic tolerance. Although we did not quantify microglial activation in this study, BTP2 has been shown to inhibit both astrocytes and microglia in other pain models (Siddiqui et al., 2012; Ferreira and Schlichter, 2013). Future studies could investigate whether microglial modulation contributes to BTP2’s reversal effect on established tolerance.

Previous studies have shown that the spinal astrocytic mitogen-activated protein kinase (MAPK), including ERK, is involved in morphine analgesic tolerance (Wang et al., 2010; Berta et al., 2013). It has also been indicated that SOCE is involved in ERK activation in non-neuronal cells (Voelkers et al., 2010). And our previous study demonstrated that intrathecal administration of BTP2 could suppress activation of spinal ERK in rats with neuropathic pain (Qi et al., 2016). We postulated that BTP2 may reduce ERK activation in morphine analgesic tolerance. In agreement with our hypothesis, our present study demonstrates that intrathecal BTP2 dose-dependently reduced the upregulated level of p-ERK in the spinal astrocytes. Similar to our findings, Gao and colleagues (Gao et al., 2015) found that BTP2 inhibited collagen-induced arthritis pain by reducing p-ERK in the spinal cord *in vivo*. Activation of ERK in the astrocytes was found to associate with the expression of proinflammatory cytokines in morphine tolerance. A previous study has shown that chronic morphine could induce the activation of ERK, which was mostly expressed in astrocytes, in the lumbar spinal cord, and the activation of ERK is a key regulator of the expression of TNF-α and Il-1β (Wang et al., 2009). These findings suggest that the mechanisms underlying the role of BTP2 in attenuating morphine analgesic tolerance seem by suppressing the activation of ERK, which with closely linked to astrocyte activation and TNF-α as well as Il-1β production. Although we did not measure STIM1/Orai1 expression in this study, our findings are consistent with prior evidence that SOCCs are functionally expressed in spinal astrocytes and regulate cytokine release. The observed effects of BTP2 further support the involvement of SOCC signaling in morphine tolerance.

It is now established that chronic morphine-induced proinflammatory cytokines, such as TNF-α and IL-1β, contribute to tolerance (Eidson and Murphy, 2019; Hutchinson et al., 2008). The proinflammatory cytokines could sensitize nearby neurons, increase the release of pain-relevant neurotransmitters, enhance the number and conductivity of AMPA and NMDA glutamate receptors, decrease astrocytic glutamate transporter proteins, and down-regulate GABA receptors, resulting in an overall enhanced neuronal excitability and amplification of pain (Watkins et al., 2005; Ji et al., 2013). Pharmacological blockade of these pro-inflammatory cytokines could enhance morphine effectiveness and attenuate tolerance (Hutchinson et al., 2008; Hameed et al., 2010). It has been shown that the proinflammatory effects of morphine are mediated through the innate immune Toll-like receptor 4 (TLR4). TLR4 was found expressed on microglia and astrocytes (Kielian, 2006; Shen et al., 2016). Morphine binds to the TLR4 accessory protein myeloid differentiation protein 2 (MD-2) and induces TLR4/MD-2 oligomerization and initiates an inflammatory response was similar to the classic TLR4 ligand lipopolysaccharide (LPS) (Hutchinson et al., 2011; Wang et al., 2012). Interestingly, a previous study showed that the SOCCs blocker BTP2 could attenuate LPS-induced lung injury (Gandhirajan et al., 2013), further supporting the anti-inflammatory role of BTP2. Our results demonstrated that 2 or 10 nmol intrathecally decreased the production of TNF-α and IL-1β, suggesting the ability of BTP2 to reduce pro-inflammatory cytokines secretion could play a role in alleviating morphine tolerance.

SOCCs are highly plasma membrane channels that can be activated by the depletion of endoplasmic reticulum Ca^2+^ stores, and SOCE is a major mechanism of Ca^2+^ influx in immune cells. Recently, there has been increasing interest in the potential role of SOCCs in the modulation of pain. It has been demonstrated that STIM1 and Orai1 proteins, the key components of SOCCs, are expressed in the spinal cord dorsal horn neurons (Xia et al., 2014), dorsal root ganglion neurons (Gemes et al., 2011), as well as microglia (Michaelis et al., 2015) and astrocytes (Gao et al., 2016). Although we did not measure STIM1/Orai1 expression in this study, our findings are consistent with prior evidence that SOCCs are functionally expressed in spinal astrocytes and regulate cytokine release (Gao et al., 2016). The observed effects of BTP2 further support the involvement of SOCC signaling in morphine tolerance. It has also been indicated that SOCCs play a role in chronic pain at the spinal cord level, such as neuropathic pain (Qi et al., 2016; Gao et al., 2013) and inflammatory pain (Gao et al., 2015), and BTP2 administration could attenuate these types of pain (Qi et al., 2016; Gao et al., 2015; Gao et al., 2013). Furthermore, previous studies have also proposed that morphine analgesic tolerance and neuropathic pain have similar cellular mechanisms involving both glia activation and inflammatory cytokines production (Raghavendra et al., 2002; DeLeo et al., 2004), so it is reasonable to speculate that BPT2 could interfere with tolerance to morphine analgesia. In agreement with our hypothesis, we found that the administration of BTP2 attenuated morphine analgesic tolerance, suggesting SOCCs are involved in the development of morphine tolerance. We acknowledge that BTP2 may have off-target effects, but it remains one of the most widely used pharmacological inhibitors of SOCCs *in vivo* (Prakriya and Lewis, 2015; Gao et al., 2016). Future studies using genetic approaches could further confirm the SOCC-specific mechanisms.

In summary, the present study indicates that the SOCCs inhibitor BTP2 not only prevents the development of morphine antinociceptive tolerance but also partially reverses the established tolerance. The mechanisms of these actions appear to be mediated by decreasing astrocytic ERK activation and reducing pro-inflammatory cytokines TNF-α and IL-1β in the spinal cord. These findings suggest that SOCCs may play a role in morphine analgesic tolerance, and SOCCs could be considered as a therapeutic target for such tolerance. While this study provides proof-of-concept, future investigations should include dose–response analyses, long-term outcome assessments, and direct mechanistic studies to enhance translational relevance.

## Data Availability

The original contributions presented in the study are included in the article/supplementary material, further inquiries can be directed to the corresponding author.
